# Biliary Sepsis Caused by Burkholderia cepacia: A Rare Presentation

**DOI:** 10.7759/cureus.98412

**Published:** 2025-12-03

**Authors:** Lee Matthew L Ponce, Gelza Mae A Zabat

**Affiliations:** 1 Infectious Disease, St. Luke's Medical Center, Quezon City, PHL

**Keywords:** biliary sepsis, burkholderia cepacia, cholangitis, immunocompromised, nosocomial biliary tract infection

## Abstract

*Burkholderia cepacia* is notorious for causing nosocomial pneumonia in immunocompromised patients, while extrapulmonary infections remain uncommon. We report the case of a 53-year-old man recently diagnosed with lung adenocarcinoma with liver and spine metastases who initially presented with obstructive jaundice. Laboratory workup revealed elevated alkaline phosphatase, alanine aminotransferase, and direct hyperbilirubinemia. Baseline chest X-ray showed a soft tissue density in the left lower lobe, and dynamic liver CT demonstrated multiple liver nodules suspicious for metastasis. He underwent endoscopic retrograde cholangiopancreatography with stent insertion. Post-procedure, the patient developed a fever. He was managed as a case of cholangitis and started on piperacillin-tazobactam. Blood cultures grew *B. cepacia*, which was sensitive to meropenem, levofloxacin, and cotrimoxazole; therefore, antibiotics were shifted to meropenem. Despite this, he remained febrile. Magnetic resonance cholangiopancreatography showed dilatation of both the hepatic and common bile ducts with the stent in place. The team proceeded with percutaneous transhepatic biliary drainage. Bile cultures again grew *B. cepacia* with the same sensitivities. Repeat blood cultures remained positive, so levofloxacin was added to meropenem. Two days after starting the combination regimen, the patient became afebrile. Subsequent blood cultures showed clearance. He was discharged in stable condition with two weeks of oral levofloxacin. *B. cepacia* should be considered a possible causative agent of nosocomial biliary tract infections in immunocompromised patients.

## Introduction

*Burkholderia cepacia* is an aerobic, motile, gram-negative bacillus that is inherently resistant to many antibiotic classes, posing a challenge in management. It has several virulence factors that contribute to disease, including cell receptors (mainly CK13 receptors in respiratory epithelium) that enable host-cell binding, siderophores that allow utilization of host iron stores, and the ability to form biofilms [1]. *B. cepacia* is notorious for causing respiratory infections, particularly in immunocompromised patients and those with cystic fibrosis. In fact, in the absence of cystic fibrosis, *B. cepacia* bacteremia is usually associated with pneumonia. Recommended treatment options include broad-spectrum antimicrobials such as ceftazidime, meropenem, and minocycline [1].

Although *B. cepacia* is well recognized as a cause of nosocomial pneumonia, reports of extrapulmonary infections remain scarce [1]. This paper describes a case of biliary sepsis caused by this organism.

This case was previously presented as a poster abstract at the 20th Asia Pacific Congress of Clinical Microbiology and Infection (APCCMI) held on November 2-4, 2025, in Bangkok, Thailand.

## Case presentation

A 53-year-old Filipino man with no known comorbidities presented with a six-month history of non-productive cough without fever or weight loss. His symptoms persisted, and after four months, he developed back pain and jaundice. The severity of his back pain prompted consultation with an orthopedic surgeon, who advised a thoracic spine MRI. Imaging revealed compression deformities of the T2 vertebra and lytic lesions suspicious for bone metastases. He was subsequently advised to be admitted for further workup. Regarding antibiotic exposure, he had recently completed a course of cefuroxime for presumed pneumonia. He had no recent travel, hospitalizations, or sick contacts. He denied previous pulmonary tuberculosis or known exposures. Family history was notable for cervical cancer on the maternal side. He was a non-smoker but had significant secondhand smoke exposure. He did not consume alcohol.

On examination, vital signs were within normal limits. Pertinent findings included icteric sclerae, jaundice, and crackles over the left mid to lower lung fields. The abdomen was flat, soft, and nontender. The liver was not palpable, and no fluid wave was appreciated. 

**Figure 1:**
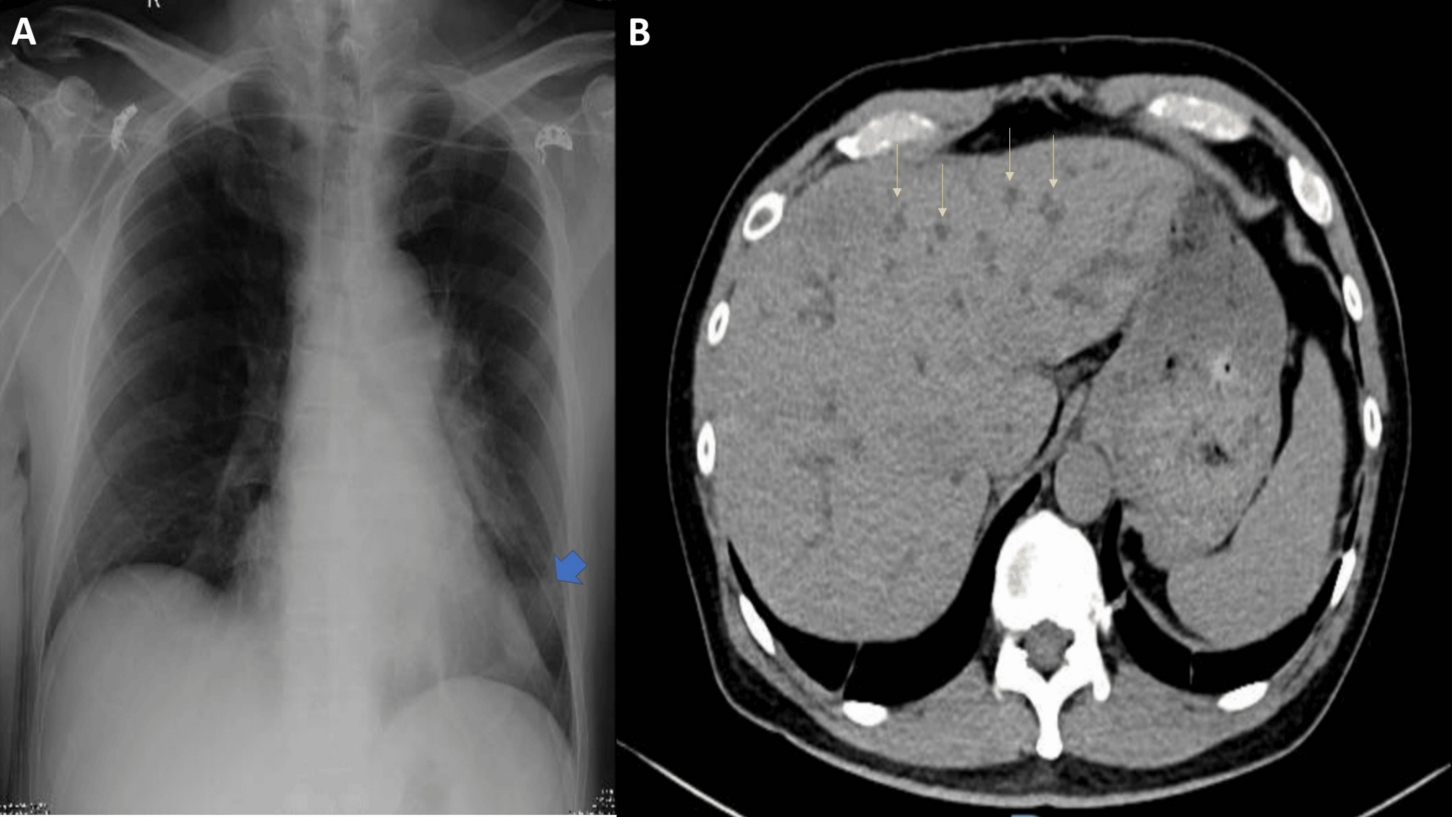
(A) Chest X-ray at admission showing a left lower lobe mass. (B) Dynamic liver CT scan demonstrating multiple nodules in the liver parenchyma. Blue arrow: left lower lobe lung mass. Yellow arrows: hepatic nodules.

Notable laboratory tests revealed leukocytosis of 17.13 × 10³/µL (normal 4.23-9.07 × 10³/µL) with neutrophilic predominance at 0.66 (normal 0.34-0.65). Liver function tests were elevated, with alanine transaminase at 111 U/L (normal 13-69 U/L), alkaline phosphatase at 862 U/L (normal 44-147 U/L), and hyperbilirubinemia characterized by a total bilirubin of 5.87 mg/dL (normal 0.1-1.2 mg/dL) and direct bilirubin of 4.82 mg/dL (normal <0.3 mg/dL). Baseline chest X-ray showed a soft tissue density in the left lower lung (Figure 1A). A dynamic liver CT scan demonstrated irregular hypodense nodules scattered throughout both hepatic lobes, suggestive of metastases (Figure 1B). Given these findings, a percutaneous biopsy of the lung mass was performed, which confirmed adenocarcinoma. Cultures and tuberculosis studies of the lung tissue were negative. On the second hospital day, he underwent endoscopic retrograde cholangiopancreatography (ERCP) with sphincterotomy and stent placement to address obstructive jaundice. 

He developed fever episodes after the procedure and was managed as a case of cholangitis, initially started on piperacillin-tazobactam. Blood cultures later grew *B. cepacia*, which was sensitive to meropenem, levofloxacin, and cotrimoxazole; therefore, the antibiotics were shifted to meropenem. Despite the change in treatment, he remained febrile. Magnetic resonance cholangiopancreatography was performed and showed dilatation of both the hepatic and common bile ducts, although the previously inserted stent was still in place. The team then proceeded with percutaneous transhepatic biliary drainage. Bile cultures also grew *B. cepacia* with the same sensitivity profile. Repeat blood cultures, taken despite adequate antibiotic dosing, remained positive for the same organism, so levofloxacin was added to meropenem. Two days after starting the combined regimen, the patient became afebrile. A subsequent set of blood cultures eventually showed clearing. He was discharged in stable condition with oral levofloxacin for two weeks as home therapy.

## Discussion

Ascending cholangitis is a serious intra-abdominal infection, with systemic infection rates reported as high as 20%. Obstruction of the biliary tract remains the major pathophysiologic mechanism, leading to impaired bile flow and bacterial proliferation within the biliary system [2]. In a study of 205 patients with recurrent ascending cholangitis from two institutions in Romania and France, Cozma et al. found that gram-negative organisms remained the predominant etiologic agents [2]. Similarly, Li et al., in their review of 503 cases from a single institution in China, identified *Enterococcus* spp. and gram-negative rods as the leading causes of cholangitis [3]. Across both studies, the most commonly isolated pathogens were *Escherichia coli*, *Enterococcus* spp., *Pseudomonas* spp., and *Klebsiella* spp. [2,3].

*B. cepacia* is primarily known as a cause of nosocomial pneumonia, and reports of extrapulmonary infections remain limited [1]. One case series from a tertiary hospital in Lebanon described 44 infections due to *B. cepacia*, most of which were catheter-related bloodstream infections (38.6%), followed by skin and soft tissue infections (36.4%) and vertebral osteomyelitis (18.2%); only one case of *B. cepacia* peritonitis was reported [4].

There are isolated reports of *B. cepacia* causing intra-abdominal infections. Mukhopadhyay et al. described a 56-year-old man with chronic obstructive pulmonary disease who was found to have a liver abscess, with aspirate cultures growing *B. cepacia* [5]. Ohji et al. reported a 57-year-old woman presenting with progressive jaundice who underwent ERCP for a biliary tumor causing obstruction. Initial bile cultures were negative, but she developed post-procedure fever, and repeat blood and bile cultures grew *Burkholderia contaminans*, a member of the *B. cepacia* complex [6].

Several mechanisms have been proposed to explain how *B. cepacia* causes extrapulmonary infections. Ohji et al. noted that the organism is a frequent contaminant of medical devices such as central venous catheters and urinary catheters [6]. Our patient did not have any of these devices inserted during hospitalization, and serial chest X-rays did not show evidence of pneumonia, making the respiratory tract an unlikely source. A common feature between our case and that of Ohji et al. is the development of cholangitis after ERCP with stent insertion. In Ohji’s case, only post-procedure bile cultures turned positive, confirming nosocomial acquisition. Their institution investigated possible endoscopy-related infections but found none, as decontamination protocols were strictly followed [6]. Similarly, no endoscopy-related infections were identified at our hospital, and decontamination procedures adhered to institutional standards. Bile cultures were not sent during the initial ERCP in our patient because he did not exhibit symptoms of cholangitis at that time. Given the chronology, onset of fever following ERCP with stent placement, a nosocomial biliary infection is most likely.

*B. cepacia* is well known for its intrinsic resistance to multiple antimicrobial classes [1]. In our case, the isolate was susceptible to meropenem, cotrimoxazole, and levofloxacin. The organism in the case reported by Ohji et al. was susceptible only to carbapenems and cotrimoxazole, while the isolate in the report by Mukhopadhyay et al. was sensitive only to ciprofloxacin and meropenem. Both cases were initially treated with broad-spectrum antibiotics (cefoperazone-sulbactam and meropenem, respectively) and were later shifted to narrower agents once sensitivities were known [5,6]. In our case, meropenem was started once culture data became available, and levofloxacin was added when bacteremia persisted.

## Conclusions

We describe one of the few reported cases of *B. cepacia* bacteremia secondary to a biliary tract infection. Although this organism is best known for causing pulmonary infections due to its multiple virulence factors, existing literature suggests that contamination of medical devices may also serve as a mechanism for biliary tract involvement. This case highlights the importance of considering *B. cepacia* as a potential pathogen in immunocompromised patients who develop nosocomial biliary tract infections.
